# Phylogenetic analysis of β-xylanase SRXL1 of *Sporisorium reilianum* and its relationship with families (GH10 and GH11) of Ascomycetes and Basidiomycetes

**DOI:** 10.1038/srep24010

**Published:** 2016-04-04

**Authors:** Jorge Álvarez-Cervantes, Gerardo Díaz-Godínez, Yuridia Mercado-Flores, Vijai Kumar Gupta, Miguel Angel Anducho-Reyes

**Affiliations:** 1 Universidad Politécnica de Pachuca. Zempoala, Hidalgo, México; 2 Laboratory of Biotechnology, Research Center for Biological Sciences, Universidad Autónoma de Tlaxcala, Tlaxcala, México; 3 Molecular Glycobiotechnology Group, Discipline of Biochemistry, National University of Ireland Galway, Galway, Ireland

## Abstract

In this paper, the amino acid sequence of the β-xylanase SRXL1 of *Sporisorium reilianum,* which is a pathogenic fungus of maize was used as a model protein to find its phylogenetic relationship with other xylanases of Ascomycetes and Basidiomycetes and the information obtained allowed to establish a hypothesis of monophyly and of biological role. 84 amino acid sequences of β-xylanase obtained from the GenBank database was used. Groupings analysis of higher-level in the Pfam database allowed to determine that the proteins under study were classified into the GH10 and GH11 families, based on the regions of highly conserved amino acids, 233–318 and 180–193 respectively, where glutamate residues are responsible for the catalysis.

Xylanases (endo-β-xylanase, EC 3.2.1.8) are enzymes that perform the hydrolysis of the glycosidic bonds of xylan, releasing xylooligosaccharides. These proteins are produced by different taxonomic groups such as algae, crustaceans, insects, yeasts, bacteria and fungi^1^,^2^,^3^,^4^,^5^. Comparison and analysis of their amino acid sequences and hydrophobicity regions have allowed classify them into *0*-glycosyl hydrolases. Within this group, xylanases, have been classified into 13 families, according to the information provided in the Carbohydrate-Active Enzymes Database (CAZy)^6^, of all of them only the GH10 and GH11 families with exclusive activities for endo-β-xylanase^5^,^7^,^8^,^9^. The difference between the two families is at sequence level and three-dimensional structure; however, retain their catalytic sites^10^,^11^. The GH10 family belongs to clan GH-A, which are proteins that are characterized by sharing a similar tertiary structure, catalytic amino acids and enzymatic mechanism are conserved, presenting a domain for catalysis of 250 to 450 amino acids with a barrel type folding (α/β)_8_ or TIM-barrel. From the biochemical standpoint, most have high molecular weight, although there are reports of low molecular weight enzymes. The values of their isoelectric points (pI) are generally alkaline between 8.0–9.5, however, some have acid values, all maintains the same three-dimensional structure. The catalytic site makes them less strict in the use of the substrate, allowing the enzyme to be active on xylooligosaccharides of low degree of polymerization^9^,^12^.

Furthermore, the GH11 family belongs to clan GH-C, which have a similar tertiary structure, catalytic amino acids and enzymatic mechanism are conserved, presenting domains for catalysis of 180 to 200 amino acids that fold into β sheet conformation curved on itself, known as β-jelly-roll. From a biochemical standpoint, they have low molecular weight and high pI values, besides being selective as to the use of substrate with high catalytic activity^8^,^10^,^11^,^12^,^13^.

Both families use the same double displacement catalytic mechanism with retention of anomeric configuration, which involve two highly conserved glutamate residues within the active site, one of them acting as acid/base catalyst and the other as a nucleophile^8^,^14^,^15^.

Analysis of amino acid composition of these enzymes indicates that the aspartic acid, glutamic acid, glycine, serine and threonine are presented in a higher percentage^15^.

Xylanase of GH10 and GH11 families have been described in different Ascomycetes and Basidiomycetes, where actively involved in the degradation of substrates where they grow. Ascomycetes genera such as *Trichoderma, Aspergillus, Penicillium, Fusarium, Magnaphorte, Sclerotinia, Cochliobolus, Stenocarpella,* secrete xylanases of both families. In this case the first three, correspond to industrially important fungi and the last five are plant pathogens, where these enzymes are involved in the degradation of hemicellulose from plant cell wall helping to colonization and nutrition during infection^16^,^17^,^18^,^19^,^20^,^21^,^22^. Basidiomycetes that traditionally have been studied are genera of *Trametes, Pleurotus* and *Phanerochaete* by their ability to degrade lignocellulose where xylanases play an important role^23^,^24^,^25^. The production of these enzymes in plant pathogens has been described in *Ustilago maydis, Magnaphorte grisea, Fusarium oxysporum and Sclerotinia sclerotiorum*^17^,^20^,^21^,^26^ and in *Sporisorium reilianum* where a xylanase called SRXL1 has been purified and characterized biochemically^27^. The peptide sequence of the purified protein showed that corresponds to sr14403 gene deposited in the database of the genome of the fungus, which encodes an endo-β-1,4-xylanase. The similarity analysis with other proteins indicates that this enzyme belonging to the family 10 of glycosyl hydrolases^27^,^28^.

This paper proposes a hypothesis that allows knowing the phylogenetic relationship between SRXL1 xylanase and xylanases of GH10 and GH11 families of Ascomycetes and Basidiomycetes using *Trametes versicolor* as fossil record to estimate divergence times of these enzymes, as several authors have proposed that the alignment of sequences of the proteins could reveal useful information about their functional and evolutionary relationships, since it is considered that the amino acid residues that occupy equivalent positions may share a common ancestor or present the same biological role^15^.

## Results

### Selection of the xylanase sequences

Based on the results of BLAST analysis, 83 protein sequences corresponding to β-xylanases of fungi Ascomycetes and Basidiomycetes were selected (65 and 18, respectively), which exhibit similarity to the sequence of the SRXL1 xylanase of *S. reilianum*. Access numbers for each protein and the name of the microorganism to which they belong displayed below.

#### Ascomycetes

*Magnaporthe oryzae* (G4NA54, G4MVY2, G4N696, G4MWS3, G4MLU0, G4NIM7, G4NBN8), *Magnaporthe grisea* (Q92245, P0CT48, Q92244, Q8JIY4, Q01176), *Fusarium oxysporum* (F9F5R3, Q9C1R1, Q9C1R2, F9FP27, F9FI26, B3A0S5, O59938), *Fusarium graminearum* (Q7ZA57)*, Cochliobolus carbonum* (Q06562, Q00350, Q6GXE5), *Thermomyces lanuginosus* (F8UV78, O43097), *Trichoderma reesei* (p36217, P36218), *Trichoderma harzianum* (P48793), *Aspergillus niger* (P55330, P55329, Q12550, Q6QJ75, Q12549, G3Y866, C5J411), *Emericella nidulans* (P55333, P55332), *Aurobasidium pullulans* (Q9UW17, Q12562, Q96TR7, Q2PGV8), *Sclerotinia sclerotiorum* (A7EXM7, A7EQZ6), *Paecilomyces variotii* (P81536), *Trichoderma virens* (G9NBD2), *Penicillium purpurogenum* (Q9P8J1), *Penicillium oxalicum* (E1B2N4, HQ157197), *Talaromyces aerugineus* (G8ZAH1), *Thermoascus aurantiacus* (P23360), *Aspergillus kawachii* (P33559, GAA92552, JT0608), *Aspergillus oryzae* (O94163, Q96VB6), *Aspergillus fumigatus* (Q0H904), *Aspergillus versicolor* (A2I7V1), *Aspergillus sojae* (Q9P955, BAA92882), *Aspergillus terreus* (Q4JHP5, AFD63136, H9BYX9, Q0CBM8), *Aspergillus aculeatus* (O59859), *Penicillium crysogenum* (B6F253), *Penicillium citrinum* (BAG12101, B1B533), *Penicillium simplicissimum* (P56588), *Penicillium canescens* (Q5S7A8).

#### Basidiomycetes

*Coprinopsis cinerea* (A8P570, A8P8F), *Ustilago hordei* (I2FWP8), *Sporisorium reilianum* (E7A3D3), *Schizophyllum commune* (D8Q1J8), *Trametes versicolor* (EIW54190), *Punctularia strigosozonata* (EIN11616), *Phanerochaete chrysosporium* (Q9HEZ2, Q9HEZ1, AAG44993, G0ZCU2, Q9HEZ0, Q9HEY9), *Ustilago maydis* (Q4P0L3), *Pleurotus ostreatus* (B0FX60).

### Analysis of the sequences

The Pfam database allows to identify the position of the catalytic domains of each of the sequences that make them belong to the GH10 and GH11 families of glycosyl hydrolases, where were obtained 46 and 38 respectively, and the position of glutamate residues responsible for catalysis, which were located in said region.

**Figure 1:**
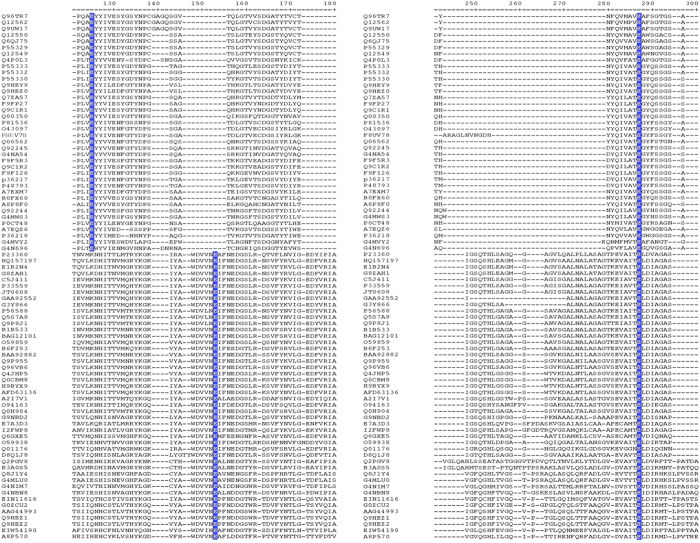
Alignment of amino acid sequence of the active site region of the family GH11 xylanases: *M. oryzae* (G4NA54, G4MVY2, G4N696, G4MWS3), *M. grisea* (Q92245, P0CT48, Q92244), *F. oxysporum* (F9F5R3, Q9C1R1, Q9C1R2, F9FP27, F9FI26), *F. graminearum* (Q7ZA57), *C. carbonum* (Q06562, Q00350), *T. lanuginosus* (F8UV78, O43097), *T. reesei* (p36217, P36218), *T. harzianum* (P48793), *A. niger* (P55330, P55329, Q12550, Q6QJ75, Q12549), *E. nidulans* (P55333, P55332), *A. pullulans* (Q9UW17, Q12562, Q96TR7), *S. sclerotiorum* (A7EXM7, A7EQZ6), *P. variotii* (P81536), *C. cinerea* (A8P8F0), *P. chrysosporium* (Q9HEZ0, Q9HEY9), *U. maydis* (Q4P0L3), *P. ostreatus* (B0FX60). And family GH10: *M. oryzae* (G4MLU0, G4NIM7, G4NBN8), *M. grisea* (Q8JIY4, Q01176), *F. oxysporum* (B3A0S5, O59938), *C. carbonum* (Q6GXE5), *A. niger* (G3Y866, C5J411), *A. pullulans* (Q2PGV8), *T. virens* (G9NBD2), *P. purpurogenum* (Q9P8J1), *P. oxalicum* (E1B2N4, HQ157197), *T. aerugineus* (G8ZAH1), *T. aurantiacus* (P23360), *A. kawachii* (P33559, GAA92552, JT0608), *A. oryzae* (O94163, Q96VB6), *A. fumigatus* (Q0H904), *A. versicolor* (A2I7V1), *A. sojae* (Q9P955, BAA92882), *A. terreus* (Q4JHP5, AFD63136, H9BYX9, Q0CBM8), *A. aculeatus* (O59859), *P. crysogenum* (B6F253), *P. citrinum* (BAG12101, B1B533), *P. simplicissimum* (P56588), *P. canescens* (Q5S7A8), *C. cinerea* (A8P570), *U. hordei* (I2FWP8), *S. reilianum* (E7A3D3), *S. commune* (D8Q1J8), *T. versicolor* (EIW54190), *P. strigosozonata* (EIN11616), *P. chrysosporium* (Q9HEZ2, Q9HEZ1, AAG44993, G0ZCU2). Gaps are indicated by a hyphen (–). A standard numbering system for the active site is included up of each block of sequences. The motives conserved in xylanases are indicated in blue that corresponds to the glutamates of the active site.

Figure 1 shows the alignment of conserved peptide sequences corresponding to the region where the glutamates responsible for catalysis were found, differences in the position of the first catalytic glutamate was observed between the two families of xylanases, as it is positioned at the amino acid number 125 for GH11 family and 153 for GH10 family. The second glutamate was aligned in the same position at the amino acid 287 in both families. This same analysis showed that all the analyzed sequences show similarity and conserved sites among them.

### Phylogenetic analysis

**Figure 2:**
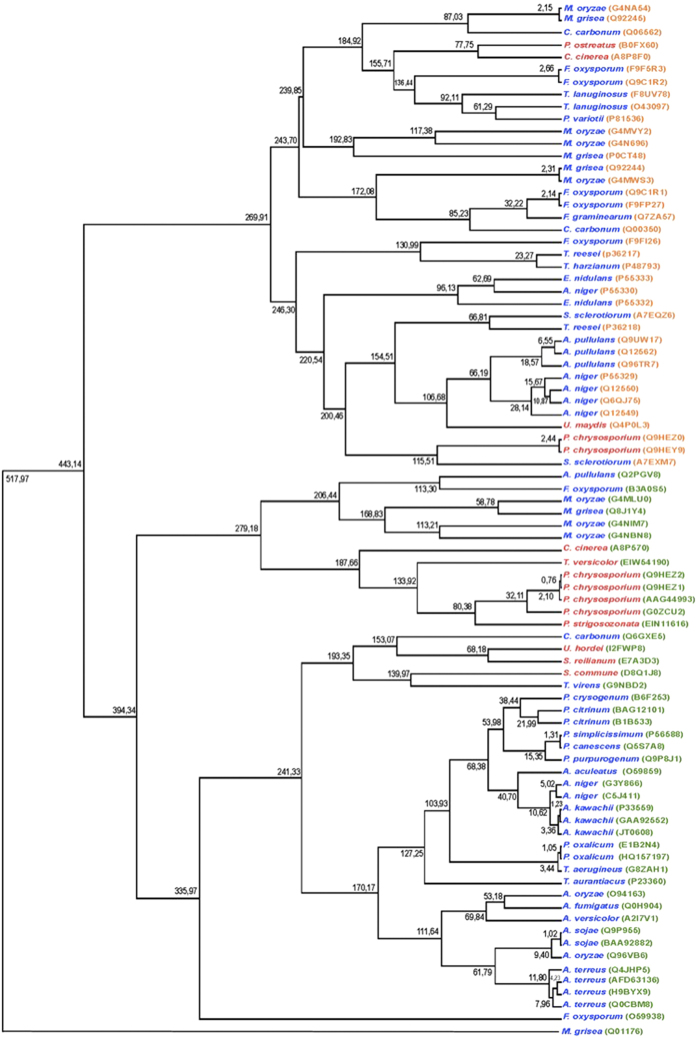
Phylogenetic analysis of the amino acid sequences of xylanases from Ascomycetes (blue letters) and Basidiomycetes (red letters) of the families GH10 (Access codes on green) and GH11 (Access codes on orange). The numbers located in the clades indicate the estimated time of divergence for enzymes in millions of years (Myr).

Phylogenetic analysis allowed to build a tree with 84 xylanase sequences where the xylanase of *M. grisea* (Q01176 sequence) belonging to the GH10 family was used as outgroup, having an estimated time of ~517 Myr. In phylogenetic construction, two groups were observed, identifying sequences belonging to the GH10 and GH11 families. The group of the family GH10, shows a grouping of the protein sequences depending to the phylum of the fungi which produce these enzymes, however, it was observed that within the group of basidiomycetes were included the xylanases of *C. carbonum* (Q6GXE5 sequence) and of *T. virens* (G9NBD2 sequence) which are ascomycetes. Xylanase of the basidiomycete *S. reilianum* (E7A3D3 sequence) was grouped with the xylanases of fungal pathogens such as *U. hordei* (Basidiomycete) and *C. carbonum* (Ascomycete). The clade of *U. hordei* and *S. reilianum* showed a common ancestor with estimated time of ~68.18 Myr; all this clade with *C. carbonum* has a common ancestor with an estimated time of ~153.06 Myr, furthermore, these proteins have a common ancestor with *S. commune* and *T. virens*, with a divergence time of ~193.35 Myr. Furthermore, the xylanase of *T. versicolor* was grouped with xylanases of *P. chrysosporium, P. strigozonata* and *C. cinerea* with estimated time of ~187.65 Myr with respect to their common ancestor. For xylanases of the family GH11, was not observed grouping that differentiates to enzymes produced by plant pathogens or those free-living. However, proteins that are produced by ascomycetes or basidiomycetes were grouped with respect to the phylum of fungus that produces them, that is to say, were identified for basidiomycetes fungi 3 groups of xylanase, the first corresponds to *P. ostreatus* and *C. cinerea*, the second to *P. chrysosporium*, while the third group was to *U. maydis*. The remaining groups corresponded to the phylum Ascomycota (Fig. 2).

### Relationship of the structure of the xylanases with respect to phylogeny

Based on the results of phylogenetic analysis of the xylanases studied, 12 and 15 sequences of GH10 and GH11 families were selected, respectively; the importance of each fungus was considered based on their phytopathogenic character and ecological or industrial importance, and the relationships that were found in the tree obtained, which is described below.

GH10 family: *S. reilianum, M. grisea* (with two sequences), *C. carbonum, U. hordei, F. oxysporum, A. sojae, A. versicolor, T. versicolor, T. virens, S. commune* and *P. chrysosporium*; GH11 family: *M. grisea* (with two sequences), *M. oryzae, F. oxysporum, A. niger* (with two sequences), *F. graminearum, P. ostreatus, C. cinereae, C. carbonum* (with two sequences), *U. maydis, E. nidulans* (with two sequences), *A. pullulans.*

**Figure 3:**
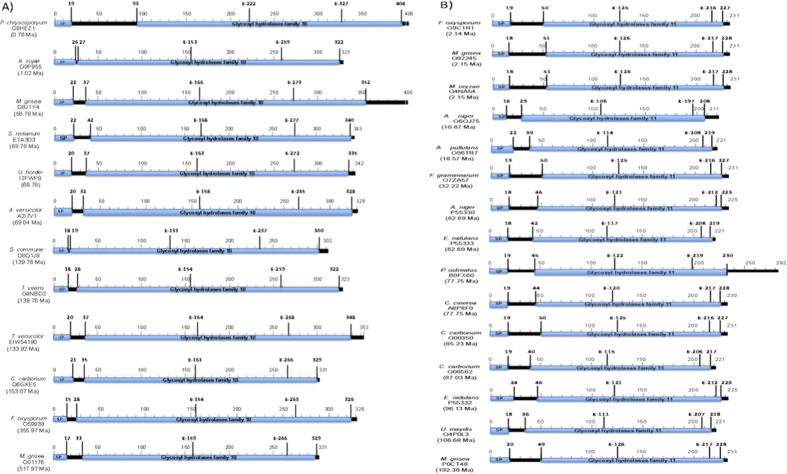
Motifs elements present in the fungal xylanases of the family GH10 (A): *P. chrysosporyum*, Q9HEZ1; *A. sojae*, Q9P955; *M. grisea*, Q8J1Y4; *S. reilianum*, E7A3D3; *U. hordei*, I2FWP8; *A. versicolor*, A2I7V1; *S. commune*, 8Q1J8; *T. virens*, G9NBD2; *T. versicolor*, EIW54190; *C. carbonum*, Q6GXE5; *F. oxysporum*, O59938; *M. grisea*, Q01176 y GH11 (B): *F. oxysporum*, Q9C1R1; *M. grisea*, Q92245; *M. oryzae*, G4NA54; *A. niger*, Q6QJ75; *A. pullulans*, Q96TR7; *F. graminearum*, Q7ZA57; *A. niger*, P55330; *E. nidulans*, P55333; *P. ostreatus*, B0FX60; *C. cinerea*, A8P8F0; *C. carbonum*, Q00350; *C. carbonum*, Q06562; *E. nidulans*, P55332; *U. maydis*, Q4P0L3; *M. grisea*, P0CT48. The numbers indicate the number of amino acid or position of signal peptide (SP). The region conserved in all proteins is shown in blue. Glutamates responsible for catalysis are indicated by the letter E followed by a number that shows its position in the sequence.

In Fig. 3, it can be seen that the signal peptide of all enzymes used for modeling has a length of 15 to 26 amino acids. The conserved region of the proteins that identify them as families GH10 and GH11 have a length of 294–315 and 177–185 amino acids, respectively. Mature proteins of GH10 and GH11 families showed 301–389 and 199–263 amino acids, respectively. Glutamate residues responsible for catalysis are shown in all sequences. Because of heterogeneity in the number of amino acids, for GH10, the positions of the first glutamate are found between amino acids 133 to 222 and the second between 263 to 327. In the GH11, the first glutamate was found at the position 106 to 126 and the second between 197 and 219. In both families, in terms of primary structure was not observed a relationship between the number of amino-acids with respect to the estimated time of appearance.

**Figure 4:**
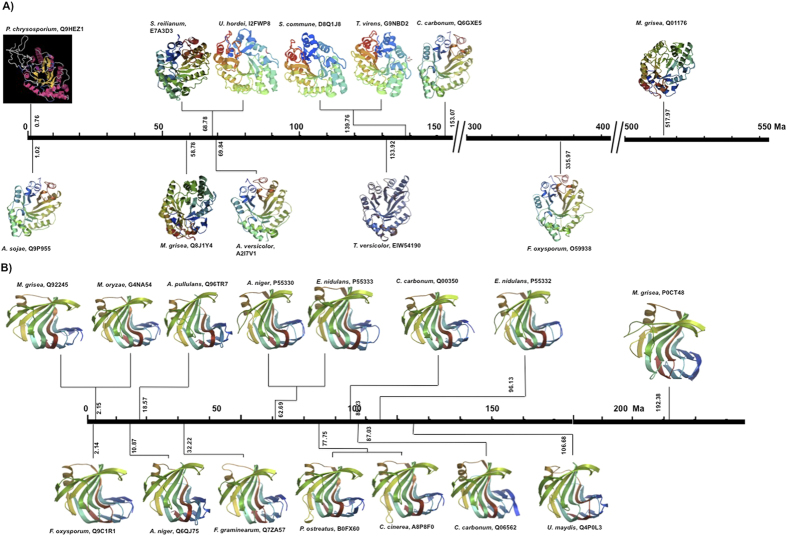
Relationship of the theoretical structure of the xylanases of GH10 family (A) *P. chrysosporyum*, Q9HEZ1; *A. sojae*, Q9P955; *M. grisea*, Q8J1Y4; *S. reilianum*, E7A3D3; *U. hordei*, I2FWP8; *A. versicolor*, A2I7V1; *S. commune*, 8Q1J8; **, G9NBD2; *T. versicolor*, EIW54190; *C. carbonum*, Q6GXE5; *F. oxysporum*, O59938; *M. grisea*, Q01176 and GH11 (B) *F. oxysporum*, Q9C1R1; *M. grisea*, Q92245; *M. oryzae*, G4NA54; *A. niger*, Q6QJ75; *A. pullulans*, Q96TR7; *F. graminearum*, Q7ZA57; *A. niger*, P55330; *E. nidulans*, P55333; *P. ostreatus*, B0FX60; *C. cinerea*, A8P8F0; *C. carbonum*, Q00350; *C. carbonum*, Q06562; *E. nidulans*, P55332; U. maydis, Q4P0L3; *M. grisea*, P0CT48. With respect to time of appearance (Myr). The modeling was carried as described in methodology.

Furthermore, protein modeling allowed to observe that all have similar characteristics in terms of their three-dimensional structure. The enzymes belonging to the family GH10 show folding type barrel with α/β lamellae being more defined in xylanases of *S. reilianum, U. hordei, S. commune* (basidiomycetes)*, T. virens* y *M. grisea* (ascomycetes), however, in the enzymes of fungi *A. sojae, A. versicolor, T. versicolor, C. carbonum* and *F. oxysporum* this structure is more elongated with respect to the above. On the other hand the protein of fungus *P. chrysosporium* although it conserves the structure of Tim-barrel, it has a substrate binding module that makes it different from the others (Fig. 4A).

Proteins of GH11 family that were modeled have a structure of roller also called β-jelly-roll, formed by lamellae β and α helix, these are joined by loops, where difference was observed in terms of the loop structure in all proteins with respect to the most ancient time corresponding to *M. grisea*. Enzymes of phytopathogenic fungi have as many loops, decreasing the length of the lamellae β with respect to other (Fig. 4B).

## Discussion

In this study the phylogenetic relationships of the xylanases produced by different fungi, including Ascomycetes and Basidiomycetes were analyzed. As reference was taken the xylanase of the GH10 family called SRXL1 of maize pathogen fungus *S. reilianum*^27^. This fungus use xylan as substrate, hydrolyzing the ß-1,4 glycosidic linkages, capacity shared with the GH11 xylanase, which was the first family of glycoside hydrolase to be classified by sequence analysis^7^.

In the analysis of protein sequences encoding xylanases were included those of phytopathogenic fungi as: *M. grisea, M. oryzae, C. carbonum, U. maydis, U. hordei, F. oxysporum, F. graminearum, S. sclerotiorum.* As well as saprophytic fungi: *P. ostreatus, A. niger, A. terreus, T. versicolor, P. chrysosporium, A. versicolor, A. sojae.* It has been described that fungi have the ability to produce hydrolytic enzymes which enable them to degrade lignocellulosic materials, accessing to sugars for use as a carbon source in their growth and reproduction. So far the characteristics of these activities have different modes of action and preference for the type of substrate^29^,^30^,^31^. Phytopathogenic fungi, cause disease in a large number of plants of agricultural interest. The interaction occurs mainly between the microorganism and plant tissues, where various mechanisms play an important part in the penetration and colonization, as in the case of production of an enzyme group capable of degrading the cell wall of the host, which is composed of lignin, cellulose and hemicellulose, this last represents in nature to 30% in plants, considered the second most abundant carbon source^15^,^32^,^33^,^34^. For degradation of hemicellulose, different types of enzymes are required, amongst which are the β-1,4-xylanases, which hydrolyze the glycosidic bonds in the xylan chain. It has been reported the biochemical characteristics, systems production and industrial application of these enzymes^2^,^4^,^35^,^36^.

In recent years, the phytopathogenic fungi have been used as a study model for the production of xylanase with possible industrial applications^30^,^37^. Also, already they have been purified and characterized xylanases from the phytopathogenic *S. sclerotiorum, F. oxysporum, M. grisea, M. oryzae, C. carbonum, S. maydis, S. reilianum,* produced in different culture media using tissue of their hosts, identifying different isoenzymes, which may present a role during the life cycle of these fungi^17^,^18^,^20^,^21^,^22^,^27^. Given the importance of these enzymes and the wide range of fungi capable of produce them, it is interesting to understand the phylogenetic relationships and the estimation of divergence times that they have with a common ancestor, which can be correlated with their status of the pathogen or saprophyte of the producing organisms. Xylanase of GH10 and GH11 families used in this study had a typical catalytic domain, this theoretical evidence is according to that obtained experimentally, where the biochemical properties of these activities are very similar, for example xylanases of *S. reilianum, M. grisea, F. oxysporum, P. chrysosporium, A. sojae, C. carbonum* of GH10 family, hydrolyse the xylan just like xylanases of *A. niger, F. oxysporum* and *S. sclerotiorum* of GH11 family^20^,^21^,^27^,^34^,^38^,^39^,^40^,^41^,^42^, this is because in addition to having conserved domains, the amino acid residues responsible for catalysis are two glutamates, which were first described in *Cellulomonas fimi*, where one acts as the nucleophile and the other as acid/base at positions 233 and 127 respectively, In this last has been observed that precedes it an asparagine residue, which is involved in hydrogen interactions with the substrate 2-hydroxyl^43^,^44^,^45^.

Liao *et al*.^46^, carried out alignment of six xylanases of different fungi, and found that the active site corresponds to glycoside hydrolases of GH10 and GH11 families. It was observed that glutamates of the active site are conserved in both families, and some of them have carbohydrate binding modules possibly to cellulose.

The CAZy database discloses that GH10 and GH11 families differ in their physicochemical properties, structure and substrate specificity^9^, it supports the separation of the xylanases tested in two families observed in the alignments and the phylogenetic tree, which relates to the amino acid composition and determining the protein structure. Phylogenetic analysis shows the relationships of different xylanases of fungi such as Ascomycetes and Basidiomycetes, with reference the xylanase of *S. reilianum* which belong to the latter group. In the work of Floudas *et al*.^47^, used the fossil record of the Boletales, Agaricales and Ascomycetes fungi to determine the geological time of lignin degradation, as a molecular clock of type Bayesian relaxed in the BEAST software, where they estimated that the divergence time of sequence gene of oxidoreductases and CAZy family for Agaricomycetes was of ~290 Myr; Agaricales of ~430 a 470 Myr; and for Ascomycetes and Basidiomycetes was estimated the time for the first Manganese peroxidase of ~295 Myr. Based on these estimated times, a organismal phylogeny (chronogram) was obtained, with time for Ascomycetes and Basidiomycetes of ~518 and ~521 Myr, respectively, so was decided to use the time to Ascomycetes, to estimate divergence times of the xylanase of this study. This could be related in some way to their metabolic activity, which could provide the conditions for these organisms could colonize and utilize the available substrates. In this case enzyme secretion was probably decisive mechanism for its establishment and propagation, so it is considered that the xylanase played an important role, which is kept up to date. The phylogenetic tree revealed the evolutionary relationship of the xylanases tested, showing two clades defined, each representing the families of glycosyl hydrolases GH10 and GH11. Xylanases forming a family or group appear to have diverged from a common ancestor, these enzymes exhibit similarity in their secondary and tertiary structure and content of amino acid residues^15^. Given the above and since the tree was constructed with xylanases produced by different genera of fungi, it can see that there are enzymes with minimal differences related to genus and species. These results are related to those obtained by Naumoff *et al*.^9^, of the phylogeny of glycoside hydrolases obtained from planctomycetes, they concluded that the generation of different clades in a tree, are the result of the frequent duplications, horizontal transfer and removing genes coding for these proteins.

Clades identified in the tree shows related xylanases depending on the phylum and its pathogenic or saprophytic character, for example highlighting the phylogenetic relationship between proteins of *M. grisea* and *M. oryzae*, and of *F. oxysporum* and *F. graminearum,* as well as of *S. reilianum, U. hordei* and *C. carbonum*, that in all cases, they are in the same clade and all are phytopathogenic. In these organisms, the degradation of the cell wall to colonize the host, is performed by the action of enzymes such as pectinases, cellulases, proteases and xylanases which have been shown to be related to the pathogenicity and virulence^30^,^48^,^49^,^50^,^51^,^52^. The xylanase of the phytopathogenic fungus *M. grisea* was the protein with the greatest divergence time, which belongs to the GH10 family, this fungus infects mainly to rice, however, it can infect grasses such as barley and wheat, by production of an enzyme complex that also allows access to the carbon source present in the cell wall. The genome sequence of this fungus, shows the presence of up to 20 genes with xylanolytic activity for different xylanase families, which suggests that these activities are essential for the life cycle and can be a determinant in setting its saprophytic or pathogenic character^42^,^53^. Something similar can be for *F. oxysporum* in which found several genes encoding xylanases, which could be related to the pathogenesis of the fungus^17^,^18^,^42^,^54^.

Xylanase of *S. reilianum* has an estimated divergence time of ~68.18 Myr, possibly related to the recording time for its host, since which have been estimated sometimes of polyploidization for angiosperms approximately of ~70 Myr, before the divergence of the main cereals^55^. Buckler and Stevens^56^ estimated that the family Poaceae (Gramineae) was originated from a common ancestor within the last 55–70 Ma based on fossil evidence. It has been demonstrated that different isolates of phytopathogenic fungi have high activity in the degradation of xylan and untreated biomass, the preference for the use of substrates to depolymerize depends on the preference of the pathogen by the host either mono- or dicotyledonous, this makes it efficient digestion due to specialization of enzymes that exert this action^30^.

Most of the three-dimensional structures of xylanase are mainly families GH10 and GH11, this last have fold “jelly-roll” with large sheets ß and one α-helix, like the palm of a hand partially closed, where the active site is formed by the slit in the shape of fingers and thumb^57^. The family GH10, shows a structure of a barrel, observing a large radius with shape of loop^58^. These structures were maintained on the modeled proteins in the study of both families. Interestingly, the xylanase of basidiomycete fungus *P. chrysosporium* has the shortest divergence time, presenting a module substrate binding. It is known that this fungus type is excellent degrading of wood^59^,^60^. In some way efficiency is defined by the ability of enzymes to degrade the polymers that have biomass, where the conditions that favoring catalysis, gives advantages to the fungus to establish itself in a particular ecological niche. The phylogenetic relationship of xylanase SRXL1 of *S. reilianum* with the xylanases analyzed in this paper shows a monophyly and a relationship is observed with respect to their status as plant pathogens or saprophytic fungi, in this case the functionality of these enzymes is related to its adaptation to their ecological niche.

## Methods

### Selection of known sequences of xylanases

To search endo-β-xylanases, the SRXL1 xylanase sequence of *S. reilianum* was used as reference, taking the access number E7A3D3 of UniProtKB database and access number CBQ73812.1 of GeneBank database, which was subjected to a BLAST analysis in the public databases of UniProtKB (www.uniprot.org) and NCBI (www.ncbi.nlm.nih.gov).

### Analysis of the sequences

To determine the type of family of each of the sequences obtained, the position of glutamate residues of the active site and the highly conserved motifs of GH10 and GH11 families was used, the Hidden Markov Models (HMMs) algorithm of Pfam 28.0 database was used (www.pfam.xfam.org)^61^. The resulting sequences were edited manually to remove the amino acids that were not distinctive of families under study.

Once selected the highly conserved regions of each protein, a multiple alignment was done using the MAFFT v7.058b program (http://mafft.cbrc.jp/alignment/software/), which implements the Fast Fourier Transform (FFT), this algorithm optimizes protein alignments based on the physicochemical properties of amino acids^62^. Subsequently, a manual editing considering the glutamate residue present in the active site of both xylanase families was done, which were used for phylogenetic analysis.

### Phylogenetic analysis

Phylogenetic analysis was performed with the BEAST v1.8.0 program (http://www.beast2.org)^63^, using the Bayesian algorithm and using a model of amino acid substitution by the maximum-likelihood method WAG (Model Whelan And Goldman)^64^, as well as a model of Gamma Heterogeneity sites + Invariant Sites with a number of categories Gamma 4. Model Lognormal relaxed clock (Uncorrelated) to estimate the times of divergence between the sequences was used, with an estimated time for ascomycetes ≈518 Myr, previously reported by Floudas *et al*.^47^. The analysis was performed with the Markov Chain Monte Carlo algorithm (MCMC), running 10 million generations. Q01176 sequence of *Magnaporthe grisea* was used as outgroup. The consensus of the trees was performed with the TreeAnnotator v1.7.4 program with the parameters set by default. The consensus phylogenetic tree generated was displayed with the Fig Tree v1.4.0 program (http://tree.bio.ed.ac.uk/software/figtree/). Finally the image of the tree was edited with Inkscape program (https://inkscape.org/).

### Modeling of proteins

In order to establish differences in the theoretical three-dimensional structure of the protein under study, the modeling of some xylanases of those represented in the phylogenetic tree obtained was conducted using the Protein Model Portal program (http://www.proteinmodelportal.org/)^65^, and Phyre2 (http://www.sbg.bio.ic.ac.uk/servers/phyre2/)^66^. The models obtained were visualized using Swiss-Model application (http://swissmodel.expasy.org/)^67^.

## Additional Information

**How to cite this article**: Álvarez-Cervantes, J. *et al*. Phylogenetic analysis of β-xylanase SRXL1 of *Sporisorium reilianum* and its relationship with families (GH10 and GH11) of Ascomycetes and Basidiomycetes. *Sci. Rep.* **6**, 24010; doi: 10.1038/srep24010 (2016).
